# Information and decision support needs: A survey of women interested in receiving planned oocyte cryopreservation information

**DOI:** 10.1007/s10815-023-02796-x

**Published:** 2023-04-14

**Authors:** Sherine Sandhu, Martha Hickey, Sabine Braat, Karin Hammarberg, Raelia Lew, Jane Fisher, William Ledger, Michelle Peate, on behalf of the Eggsurance Collaborative Group, F Agresta, D Lieberman, R Anderson, R Norman, R Hart, L Johnson, J Michelmore, A Parle, F Summers, C Allingham

**Affiliations:** 1 Department of Obstetrics & Gynaecology, University of Melbourne, Royal Women’s Hospital, Melbourne, Australia; 2 Centre for Epidemiology and Biostatistics, Melbourne School of Population and Global Health, University of Melbourne, Melbourne, Australia; 3 MISCH (Methods and Implementation Support for Clinical and Health) Research Hub, Faculty of Medicine, Dentistry and Health Sciences, University of Melbourne, Melbourne, Australia; 4 School of Public Health and Preventive Medicine, Monash University, Melbourne, Australia; 5 Victorian Assisted Reproductive Treatment Authority, Melbourne, Australia; 6 Reproductive Services Unit, Royal Women’s Hospital, Melbourne, Australia; 7 Global and Women’s Health Unit, School of Public Health and Preventive Medicine, Monash University, Melbourne, Australia; 8 School of Women’s and Children’s Health, University of New South Wales, Sydney, Australia; 9 Department of Reproductive Medicine, Royal Hospital for Women, Sydney, Australia

**Keywords:** Elective egg freezing, planned oocyte cryopreservation, fertility preservation, decision making, decision support, information needs

## Abstract

**Purpose:**

Identifying the information and decision support needs of women interested in receiving planned oocyte cryopreservation (POC) information.

**Methods:**

An online survey of Australian women, aged 18-45, interested in receiving POC information, proficient in English, with internet access. The survey covered POC information sources, information delivery preferences, POC and age-related infertility knowledge (study-specific scale), Decisional Conflict Scale (DCS), and time spent considering POC. Target sample size (*n*=120) was determined using a precision-based method.

**Results:**

Of 332 participants, 249 (75%) had considered POC, whilst 83 (25%) had not. Over half (54%) had searched for POC information. Fertility clinic websites were predominately used (70%). Most (73%) believed women should receive POC information between ages 19-30 years. Preferred information providers were fertility specialists (85%) and primary care physicians (81%). Other methods rated most useful to deliver POC information were online. Mean knowledge score was 8.9/14 (SD:2.3). For participants who had considered POC, mean DCS score was 57.1/100 (SD:27.2) and 78% had high decisional conflict (score >37.5). In regression, lower DCS scores were associated with every 1-point increase in knowledge score (-2.4; 95% CI [-3.9, -0.8]), consulting an IVF specialist (-17.5; [-28.0, -7.1]), and making a POC decision (-18.4; [-27.5, -9.3]). Median time to decision was 24-months (IQR: 12.0-36.0) (*n*=53).

**Conclusion:**

Women interested in receiving POC information had knowledge gaps, and wanted to be informed about the option by age 30 years from healthcare professionals and online resources. Most women who considered using POC had high decisional conflict indicating a need for decision support.

**Supplementary Information:**

The online version contains supplementary material available at 10.1007/s10815-023-02796-x.

## Introduction

Uptake of oocyte cryopreservation is rapidly increasing in high-income countries [1, 2]. In the United States (US), the annual number of cycles performed grew from around 2,700 in 2012 to 13,800 in 2018 [1]. There are also reports of increased uptake and consideration of the technique resulting from the COVID-19 pandemic [3–6]. Oocyte cryopreservation was initially introduced as a fertility preservation method for patients needing gonadotoxic cancer treatment [7]. As data on the safety and efficacy of the technique improved, its experimental label was removed [8] and the option of ‘planned oocyte cryopreservation’ (POC) became more accessible for women concerned with future age-related infertility [9].

Planned oocyte cryopreservation decisions are complex, with several factors to consider including personal circumstances and values. For example, age at the time of POC is a key predictor of success, with live birth rates per thawed-oocyte-derived embryo transfer dropping from 43% for women aged <35 years at oocyte collection to 19% for those aged 41-42 years [1]. Multiple POC cycles may also be needed for a reasonable chance of a live birth from frozen oocytes [1]. Planned oocyte cryopreservation costs are substantial and pose a barrier for many women wanting to access the service [7, 10, 11]. In addition to the costs for retrieving and freezing oocytes, there are fees for ongoing storage and to utilise frozen oocytes in the future. Ovarian stimulation and oocyte collection expose women to small yet significant health risks. Complications are rare (0.4% of cycles) [12] but considered important amongst potential POC users [11, 13–15]. Also, the risk of severe maternal morbidity from pregnancy (e.g. post-partum haemorrhage requiring blood transfusion, sepsis and cardiac failure) increases from age 30 years and is highest from 45 years onwards [16]. Finally, a 10-15 year follow up study reported that most (62%) POC patients had not accessed their frozen oocytes after storage [17]. Women who do not use their frozen oocytes may regret their decision to freeze and will eventually need to make a disposition decision. Those who do not wish or are unable to use their frozen oocytes [18–21] may also face the dilemma of needing to make a disposition decision without having achieved their reproductive goals.

The limited evidence about POC decisions suggests that women may need more information and support to guide decision-making [22–25]. For example, a US study of women who had frozen oocytes reported that 16% (*n*=33) had moderate to severe regret about pursuing the option, particularly when the information and emotional support they had received was perceived as inadequate for their decision [22]. A South Korean study of women who attended POC counselling also found that almost half their participants (*n*=40) had high decisional conflict after their visit [26]. Research performed to date on POC decisions have focussed on women who proceeded with the option [22–24]. Data from a small sample of women who decided against POC (*n*=29) showed that few regretted their decision [27], however, more research is needed to understand the experiences of those who decide against it.

Availability of comprehensive and balanced POC information is limited. Most women are informed about POC from the media or online sources including fertility clinic websites [28–30]. However, media information is often simplistic and incomplete [31], and online fertility clinic information reportedly lacks quality, transparency, and aims to persuade women towards POC [32–35]. Some may also seek POC advice from primary care physicians such as general practitioners, who may feel inadequately informed to counsel their patients about the option [36] or, from fertility clinics which have commercial conflicts [37, 38].

Given the inadequacies in existing POC information and the apparent desire for decision support, this exploratory study aimed to identify the information and decision support needs of women interested in receiving POC information.

## Methods

### Design & setting

An online cross-sectional survey in a community setting.

### Participants

Eligible participants were women aged 18-45 years, living in Australia, proficient in English, with access to the internet, and interested in receiving POC information. Those who completed their family or had frozen oocytes for medical reasons were excluded. We targeted a broad population of women interested in receiving POC information to gather perspectives from different stages of the decision-making process (e.g. pre-contemplation phase: not previously considered POC; contemplation phase: actively considering POC, and; the action and reflection phase: made their decision).

### Data source

Survey questions were developed after a review of existing literature, and with the clinical and research expertise of the authors. Estimated completion time was 10-15 minutes. Fifty-two items were covered under the following sections:

- *Participant Characteristics:* Demographics, desire for (more) children in the future (yes/no/unsure), timing for (more) children in the future (as soon as I can/when I find a partner/other), and reason for interest in POC (multiple responses from a list).
- *Information Sources:* Methods used to obtain POC information (multiple responses from a list), time spent searching for POC information (multiple responses from a list), prior consultation with an in-vitro fertilisation (IVF) specialist (yes/no) and whether POC was discussed with friends (yes/no).
- *Preferences for Information Delivery:* Views about what age POC information should be provided to women (free-text), and by whom (multiple responses from a list). Participants were also asked to rank the usefulness of eight information delivery methods (brief pamphlet, paper copy Decision Aid, online Decision Aid, written booklet, information website, DVD, consultant communication aid and website with a fertility calculator) from 1=‘most useful’ to 8=‘least useful’. After reverse scoring, an average usefulness score per method was created with higher scores indicating greater usefulness (range: 1-8). To explore any other feedback participants may have, one free-text question asked if they had any comments about POC information, resources seen or received, and why certain kinds of information may or may not be helpful.
- *Knowledge:* A 14-item purposively designed knowledge scale with three response options (true/false/don’t know) measured participants’ understanding of broad concepts relating to age-related infertility and POC including its: benefits, success rates, procedure related health risks and side-effects, and alternatives. Correct responses were summed to create a total knowledge score (range: 0-14).
- *Decisional Conflict:* The validated 10-item low-literacy Decisional Conflict Scale (DCS) measured decisional conflict, which is a state of uncertainty felt when choosing a health-related course of action (e.g. to freeze/not freeze oocytes) [39]. The scale can be used before, during, and after decision-making [40]. The four DCS sub-scales measure uncertainty, and three modifiable factors associated with uncertainty, namely feeling uninformed, unclear about personal values, and unsupported in decision-making [39]. Total scores were calculated (range: 0-100) and categorised as high (>37.5), moderate (25-37.5) and low (<25) as per the DCS user manual [39].
- *Time to Decision:* Length of time spent considering POC (free-text).

### Procedure

The survey was open from June-December 2018. It was advertised in the University of Melbourne staff newsletter, and using paid Facebook advertising by the Royal Women’s Hospital, Melbourne, and the University of Melbourne’s Psychosocial Health and Wellbeing Research Unit. Facebook advertisements targeted females aged ≥18 years in Australia. Examples of the wording used in the newsletter and social media advertisements are: *“[We] want to understand what information women need to make an informed decision about [POC]. Women aged 18-45 that are interested in [POC] information, are invited to take part in a one-off survey…”* and *“Join a study about the information and decision-making needs of women interested in [POC] information”*, respectively. All advertisements included a link to the study’s information page. No advertisements were used to market POC to women. The information page detailed the study’s aim, inclusion criteria and participation requirements. Those who wished to continue were directed to the participant information and consent form to confirm their eligibility and provide informed consent. Participants were then asked to complete the online survey. There were no incentives provided for survey completion. All participant materials (i.e. the study advertisements, information page, consent form, and survey) used the terms ‘egg freezing’ or ‘elective egg freezing’ as they have better readability than POC.

### Sample size

There was no primary outcome or hypothesis defined for this study. Therefore, the target sample size was not calculated using a power-based method, but instead a precision-based method was applied. A sample of 120 participants was ascertained to allow for a two-sided 95% confidence interval (CI) around a proportion with a margin of error (half-width) <10%, providing adequate precision of the survey results.

### Data management and statistical analysis

Participant consent and survey data were collected and managed using REDCap electronic data capture tools hosted by the University of Melbourne [41, 42].

Categorical data were summarised as counts and proportions, and continuous data as means (standard deviation, SD) or medians (25^th^ to 75^th^ percentile, IQR) if skewed.

Decisional conflict and time to decision analyses were restricted to participants who had considered POC (e.g. made their decision or were considering POC at the time of the survey). Participants who had not previously considered POC were excluded from the analyses as they had not engaged in the decision and were considered inappropriate to include.

Univariable linear regression explored factors (age, relationship status, language spoken at home, education level, medical or health-related education, consulting an IVF specialist, decision outcome/uptake of POC, number of existing children, knowledge score and prior research into POC) associated with DCS score. Associations with *p*<0.20 were included in a multivariable linear regression model.

Time to decision data were coded into months by the project coordinator. Responses from three participants who were considering POC were without a time unit. These were assumed to be months. Ordered logistic regression estimated the association between time to decision (outcome: ≤6 months, 7-12 months, 13-24 months, 25-60 months, or >60 months) and DCS score (explanatory variable). The proportional odds assumption was checked before fitting the model.

Two-sided *p*-values and 95% CIs were reported without accounting for multiple testing. Missing data were deleted listwise when deriving summary statistics by excluding those with missing values from the denominator, and when fitting statistical models by excluding those with missing values for the dependent or independent variables. For example, the sample used in the multivariable linear regression model were participants who had considered POC and provided data for all included variables.

Free-text comments were analysed using thematic analysis. Comments were coded iteratively by the study coordinator into themes by identifying key words, concepts, and reflections in accordance with the Miles & Huberman framework [43]. Participants’ comments and their corresponding codes were subsequently reviewed and verified by the study lead.

All data were analysed using Stata (v15.1) [44], excluding the free-text comments which were analysed in Microsoft Excel.

## Results

**Fig. 1:**
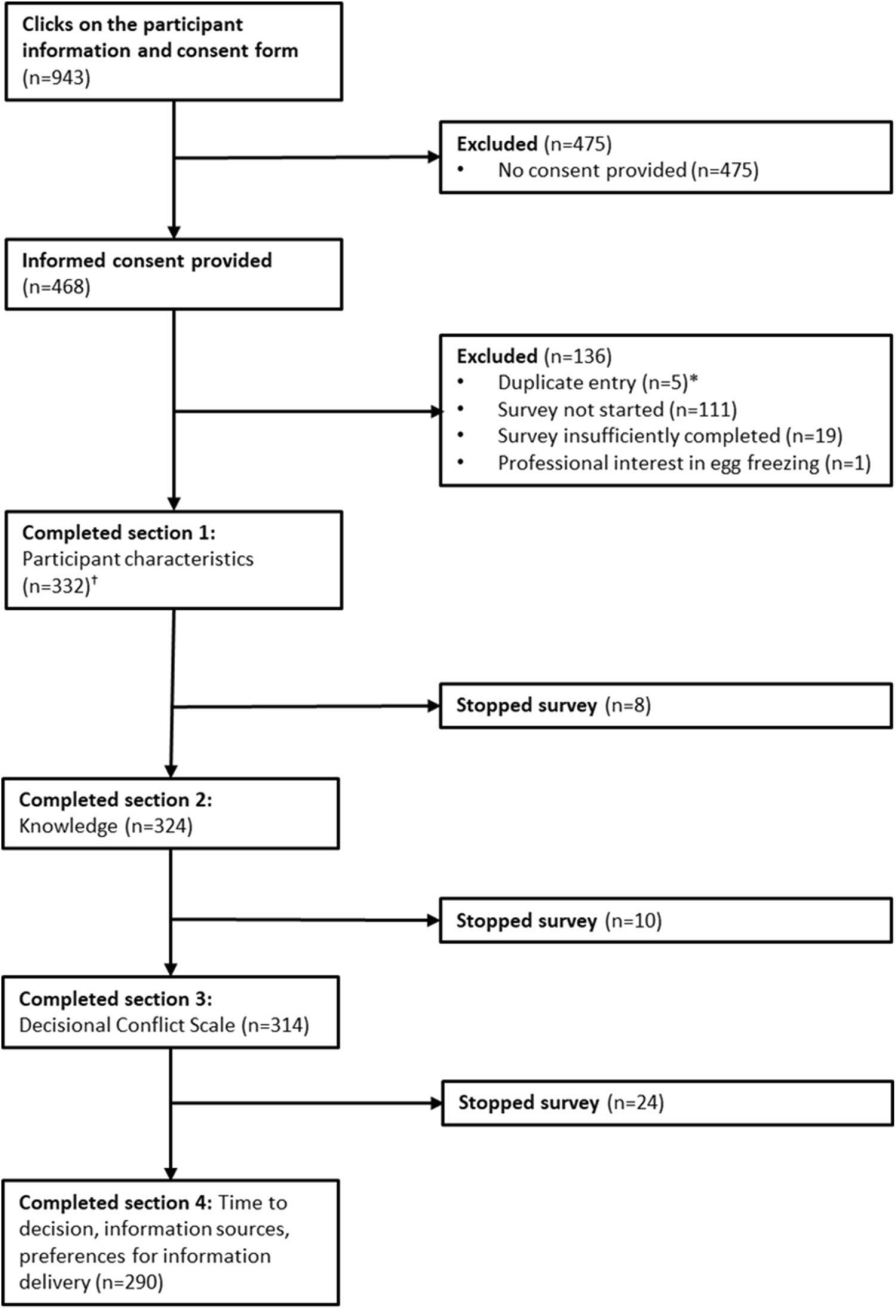
Overview of participant recruitment and survey completion. *Participants with duplicate records had both a complete and incomplete record. Complete records were retained as part of the study data, and incomplete records were removed. ^†^Data from participants who consented and completed at least the participant characteristics section of the survey (and were not otherwise excluded) were analysed

The number of eligible women who received the study invitation is unknown due to the recruitment methods used. Our participant information and consent form was clicked on 943 times. We cannot confirm if each click was by a unique and eligible person, therefore a precise response rate is unknown. Overall, 463 eligible women consented to participate. Of these, 352 (76%) started the survey and 290 (63%) completed all sections. Data from 332 (72%) women who completed at least the participant characteristics section of the survey were analysed (Fig. 1).

### Participant characteristics

Most participants were aged ≤30 years (*n*=170, 62%), single (*n*=129, 39%), university qualified (*n*=265, 80%), and working in professional occupations (*n*=247, 75%). Half (*n*=166) were educated in a medical or health-related field (Table 1). At the time of the survey, the majority (*n*=249, 75%) had considered POC. Over two-thirds (*n*=224, 68%) wished to have (more) children in the future. Of these women, most wanted to have (more) children when they found a suitable partner (*n*=74, 33%) or as soon as possible (*n*=56, 25%). Common reasons for their interest in POC were single relationship status (*n*=154, 46%), desire to invest in their future reproductive potential (*n*=94, 28%), and because of a health condition (*n*=47, 14%) (Table 2).

**Table 1:** Participant characteristics

	All (*n *= 332)	Considered POC (*n*=249)	Not previously considered POC (*n*=83)
Age*			
≤ 25 years	93 (33.9%)	66 (31.4%)	27 (42.2%)
26 to ≤ 30 years	77 (28.1%)	56 (26.7%)	21 (32.8%)
31 to ≤ 35 years	58 (21.2%)	46 (21.9%)	12 (18.8%)
36 to ≤ 40 years	34 (12.4%)	33 (15.7%)	1 (1.6%)
> 40 years	12 (4.4%)	9 (4.3%)	3 (4.7%)
Total number of responses	274	210	64
Relationship status			
Single	129 (39.0%)	106 (42.7%)	23 (27.7%)
In a committed relationship and living together, engaged, married or de facto^†^	124 (37.5%)	83 (33.5%)	41 (49.4%)
In a committed relationship but not living together	57 (17.2%)	40 (16.1%)	17 (20.5%)
In a relationship but not committed	15 (4.5%)	13 (5.2%)	2 (2.4%)
Separated/divorced	6 (1.8%)	6 (2.4%)	0 (0.0%)
Total number of responses	331	248	83
Relationship length for partnered participants*			
< 1 year	27 (14.0%)	22 (16.5%)	5 (8.3%)
1 year to ≤ 5 years	98 (50.8%)	69 (51.9%)	29 (48.3%)
> 5 years	68 (35.2%)	42 (31.6%)	26 (43.3%)
Total number of responses	193	133	60
Location: Australian state or territory*			
New South Wales	65 (19.9%)	53 (21.6%)	12 (14.6%)
Victoria	175 (53.5%)	127 (51.8%)	48 (58.5%)
Other	87 (26.6%)	65 (26.5%)	22 (26.8%)
Total number of responses	327	245	82
Location: Rural, remote or metropolitan area*			
Metropolitan	248 (75.8%)	190 (77.6%)	58 (70.7%)
Metropolitan/rural border	12 (3.7%)	10 (4.1%)	2 (2.4%)
Rural	67 (20.5%)	45 (18.4%)	22 (26.8%)
Total number of responses	327	245	82
Years living in Australia*			
< 10 years	28 (9.8%)	17 (7.9%)	11 (15.5%)
≥ 10 years	259 (90.2%)	199 (92.1%)	60 (84.5%)
Total number of responses	287	216	71
Aboriginal or Torres Strait Islander descent			
Yes	8 (2.4%)	7 (2.8%)	1 (1.2%)
No	323 (97.6%)	241 (97.2%)	82 (98.8%)
Total number of responses	331	248	83
Language spoken at home			
English	318 (95.8%)	239 (96.0%)	79 (95.2%)
Other	14 (4.2%)	10 (4.0%)	4 (4.8%)
Total number of responses	332	249	83
Highest level of education completed			
High school^‡^	37 (11.1%)	21 (8.4%)	16 (19.3%)
Trade (TAFE) certificate/diploma	30 (9.0%)	26 (10.4%)	4 (4.8%)
Bachelor degree	133 (40.1%)	98 (39.4%)	35 (42.2%)
Postgraduate diploma/degree	132 (39.8%)	104 (41.8%)	28 (33.7%)
Total number of responses	332	249	83
Studied in a medical or other health-related field			
Yes	166 (50.0%)	119 (47.8%)	47 (56.6%)
No	166 (50.0%)	130 (52.2%)	36 (43.4%)
Total number of responses	332	249	83
Employment status			
Full-time employed	193 (58.1%)	155 (62.2%)	38 (45.8%)
Part-time employed	74 (22.3%)	52 (20.9%)	22 (26.5%)
Full-time student	46 (13.9%)	30 (12.0%)	16 (19.3%)
Unemployed	8 (2.4%)	7 (2.8%)	1 (1.2%)
Other (e.g. temporarily unable to work, part-time student, self-employed, homemaker)	11 (3.3%)	5 (2.0%)	6 (7.2%)
Total number of responses	332	249	83
Occupation			
Professional	247 (74.6%)	194 (78.2%)	53 (63.9%)
Full-time student ^**§**^	46 (13.9%)	30 (12.1%)	16 (19.3%)
Other (e.g. clerk/sales, home duties, hospitality, trade, labourer)	38 (11.5%)	24 (9.7%)	14 (16.9%)
Total number of responses	331	248	83
Number of existing children			
No children	296 (89.4%)	223 (89.9%)	73 (88.0%)
At least one biological child	30 (9.1%)	21 (8.5%)	9 (10.8%)
At least one non-biological child	5 (1.5%)	4 (1.6%)	1 (1.2%)
Total number of responses	331	248	83

Data are presented as *n* (%) unless otherwise stated. POC = Planned Oocyte Cryopreservation. *Categorised from free-text responses. ^†^Original response option was ‘married/de facto’. ‘Other’ free-text responses of ‘committed and living together’ and ‘engaged’ were included in this group as they were deemed similar. ^‡^ Category created by merging response options ‘Year 11 or 12’ and ‘Year 10 or below’. ^**§**^Category created from ‘other’ free-text responses

**Table 2:** Participants’ interest, consideration and uptake of planned oocyte cryopreservation and parenting aspirations

	All (*n*=332)	Considered POC (*n*=249)	Not previously considered POC (*n*=83)
Stage of considering POC			
Have previously frozen oocytes	11 (3.3%)	11 (4.4%)	0 (0.0%)
Considered POC and made plans to go ahead with it	5 (1.5%)	5 (2.0%)	0 (0.0%)
Currently considering POC but have not made any plans	196 (59.0%)	196 (78.7%)	0 (0.0%)
Considered POC and decided not to go ahead with it	37 (11.1%)	37 (14.9%)	0 (0.0%)
Have not previously considered POC	83 (25.0%)	0 (0.0%)	83 (100.0%)
Total number of responses	332	249	83
Decision outcome/Uptake of POC			
Undecided	196 (59.0%)	196 (78.7%)	0 (0.0%)
Decided to freeze oocytes	16 (4.8%)	16 (6.4%)	0 (0.0%)
Decided not to freeze oocytes	37 (11.1%)	37 (14.9%)	0 (0.0%)
Not previously considered POC	83 (25.0%)	0 (0.0%)	83 (100.0%)
Total number of responses	332	249	83
Desire to have (more) children in the future			
Yes	224 (67.7%)	166 (66.9%)	58 (69.9%)
No	14 (4.2%)	9 (3.6%)	5 (6.0%)
Unsure	93 (28.1%)	73 (29.4%)	20 (24.1%)
Total number of responses	331	248	83
Timing to have (more) children in the future (excludes participants who did not want (more) children or were unsure)			
When suitable partner is found	74 (33.0%)	62 (37.3%)	12 (20.7%)
As soon as possible	56 (25.0%)	41 (24.7%)	15 (25.9%)
When career is established or feeling financially stable^*^	38 (17.0%)	25 (15.1%)	13 (22.4%)
When the time is right or feeling emotionally ready*	11 (4.9%)	7 (4.2%)	4 (6.9%)
In ≤5 years^*^	21 (9.4%)	15 (9.0%)	6 (10.3%)
Other	24 (10.7%)	16 (9.6%)	8 (13.8%)
Total number of responses	224	166	58
Reason for interest in POC			
It is (was) an option in case I am single when I am ready to have children ^†^	154 (46.4%)	131 (52.6%)	23 (27.7%)
It is (was) an investment for the future	94 (28.3%)	64 (25.7%)	30 (36.1%)
I have a health condition that prevents (prevented) me from having children at the moment (at that time)	47 (14.2%)	39 (15.7%)	8 (9.6%)
Other	25 (7.5%)	11 (4.4%)	14 (16.9%)
Not interested/Have not considered egg freezing^*^	7 (2.1%)	0 (0.0%)	7 (8.4%)
Unsure about having (more) children in the future^*^	5 (1.5%)	4 (1.6%)	1 (1.2%)
Total number of responses	332	249	83

Data are presented as *n* (%) unless otherwise stated. POC = Planned Oocyte Cryopreservation. *Category created from ‘other’ free-text responses. ^†^Category created by merging response options ‘I hope (hoped) to have children in the future, but I am (was) single at the time’ and ‘it is (was) an option to try and preserve my fertility in case I don’t find a partner in time’ as they were deemed similar

### Information sources

Over half the participants (*n*=162, 54%) had searched for POC information before completing the survey. For this subgroup, fertility clinic websites were the most common information source (*n*=114, 70%), followed by primary care physicians (*n*=49, 30%), and IVF specialists (*n*=44, 27%). Overall, 41 (14%) participants had consulted an IVF specialist about POC, including 13 (81%) of those who decided to freeze their oocytes, 8 (22%) of the those who decided against POC, and 20 (10%) of the those who were undecided. The length of time participants commonly spent searching for POC information was ≤6 months (*n*=55, 18%) followed by 1-2 years (*n*=47, 16%). Few (*n*=9, 3%) had spent ≥5 years searching for information. Almost half (*n*=141, 47%) had spoken with friends about POC (Table 3).

**Table 3:** Planned oocyte cryopreservation knowledge, information sources and preferences for information delivery

	All (*n*=332)	Considered POC (*n*=249)	Not previously considered POC (*n*=83)
Level of Knowledge			
Knowledge score (out of 14), mean (SD)	8.9 (2.3)	9.1 (2.2)	8.3 (2.4)
Total number of responses	324	246	78
Planned Oocyte Cryopreservation Information Sources			
Methods used to research POC^*^			
Did not research POC	139 (46.2%)	78 (33.6%)	61 (88.4%)
Looked up fertility clinic websites	114 (37.9%)	110 (47.4%)	4 (5.8%)
Spoke to a primary care physician^†^	49 (16.3%)	47 (20.3%)	2 (2.9%)
Spoke to a fertility specialist	44 (14.6%)	43 (18.5%)	1 (1.4%)
Attended an information seminar held by a fertility clinic	13 (4.3%)	13 (5.6%)	0 (0.0%)
General online research^**‡**^	10 (3.3%)	9 (3.9%)	1 (1.4%)
Other	21 (7.0%)	20 (8.6%)	1 (1.4%)
Total number of responses	301	232	69
Consulted an IVF specialist	41 (13.6%)	41 (17.6%)	0 (0.0%)
Total number of responses	302	233	69
Spoke to friends about POC	141 (47.2%)	130 (56.5%)	11 (15.9%)
Total number of responses	299	230	69
Time spent searching for POC information			
Did not look for any information	130 (43.2%)	67 (28.9%)	63 (91.3%)
Within the last 6 months	55 (18.3%)	51 (22.0%)	4 (5.8%)
6 months to 1 year ago	32 (10.6%)	30 (12.9%)	2 (2.9%)
1 to 2 years ago	47 (15.6%)	47 (20.3%)	0 (0.0%)
2 to 5 years ago	28 (9.3%)	28 (12.1%)	0 (0.0%)
> 5 years ago	9 (3.0%)	9 (3.9%)	0 (0.0%)
Total number of responses	301	232	69
Preferences for Planned Oocyte Cryopreservation Information Delivery			
Age women should be informed about POC by^§^			
≤18 years	42 (14.4%)	31 (13.7%)	11 (16.9%)
19 years to ≤30 years	214 (73.3%)	172 (75.8%)	42 (64.6%)
31 years to ≤35 years	32 (11.0%)	23 (10.1%)	9 (13.8%)
36 years to ≤40 years	4 (1.4%)	1 (0.4%)	3 (4.6%)
Total number of responses	292	227	65
Preferred providers of POC information^*^			
Fertility specialists	255 (85.0%)	200 (86.2%)	55 (80.9%)
Primary care physicians^†^	244 (81.3%)	187 (80.6%)	57 (83.8%)
Fertility counsellors	222 (74.0%)	174 (75.0%)	48 (70.6%)
Fertility nurses	207 (69.0%)	158 (68.1%)	49 (72.1%)
Presenters at a POC seminar held by a fertility clinic	144 (48.0%)	110 (47.4%)	34 (50.0%)
An independent source (e.g. not someone from a fertility clinic)	120 (40.0%)	96 (41.4%)	24 (35.3%)
Administrative staff from a fertility clinic	47 (15.7%)	39 (16.8%)	8 (11.8%)
Other	12 (4.0%)	9 (3.9%)	3 (4.4%)
Total number of responses	300	232	68
Information delivery method usefulness score, median (IQR)			
Website with accurate information	8.0 (7.0-8.0)	8.0 (7.0-8.0)	7.0 (7.0-8.0)
Total number of responses	289	222	67
Website which allows users to calculate their approximate chances of conceiving naturally given their circumstances	7.0 (6.0-8.0)	7.0 (6.0-8.0)	7.0 (6.0-8.0)
Total number of responses	288	222	66
Online interactive decision-aid	7.0 (5.0-8.0)	7.0 (5.0-8.0)	6.0 (5.0-8.0)
Total number of responses	290	223	67
Written booklet (not a decision-aid)	6.0 (4.5-7.0)	6.0 (5.0-7.0)	6.0 (4.0-7.0)
Total number of responses	288	222	66
Paper copy decision-aid	5.0 (4.0-7.0)	5.0 (4.0-7.0)	5.0 (4.0-7.0)
Total number of responses	288	221	67
Brief pamphlet	5.0 (4.0-6.0)	5.0 (4.0-6.0)	5.0 (3.0-6.0)
Total number of responses	289	222	67
Consultation communication aid (e.g. flip-chart)	5.0 (4.0-6.0)	5.0 (3.0-6.0)	5.0 (4.0-6.0)
Total number of responses	287	221	66
Take home DVD	3.0 (2.0-5.0)	3.0 (2.0-5.0)	4.0 (2.0-6.0)
Total number of responses	288	222	66

Data are presented as *n* (%) unless otherwise stated. POC = Planned Oocyte Cryopreservation, IVF = In-vitro fertilisation, SD = Standard deviation, IQR = 25th to 75th percentile. *Multiple options could be selected. ^†^Original response option was general practitioner. ^**‡**^Category created from ‘other’ free-text responses. ^**§**^Categorised from free-text responses

### Preferences for information delivery

Most participants (*n*=214, 73%) believed women should be informed about POC between ages 19-30 years. Preferred providers of POC information were fertility specialists (*n*=255, 85%), followed by primary care physicians (*n*=244, 81%), fertility counsellors (*n*=222, 74%), and fertility nurses (*n*=207, 69%). The three information delivery methods deemed most useful to support POC decisions were in online formats (Table 3).

Forty-one participants provided free-text comments. Three key themes were identified: 'a need for more detailed POC information', ‘a need to improve accessibility to POC information', and 'concerns about the commercial nature of POC’ (Table 4).

**Table 4:** Quotations illustrating the key themes derived from participant free-text comments

Theme	Illustrative Quotes
A need for more detailed POC information	*“Most sources of information are not comprehensive-they try to give a simple overview, and don’t answer the questions that matter most, or don’t answer in enough detail. They tell you about potential side effects but not the rates. They say it can be costly but don’t give you a ballpark range”*
Concerns about the commercial nature of POC	*“Fertility clinics are too financially invested in persuading women to use their services…They tend to downplay the risks of the procedure and overstate the potential benefits”*
A need for improved accessibility to POC information	“*There is very limited information available, unless you seek it out specifically from a fertility clinic…most [primary care physicians] don’t know much about the process, and it’s not something which is spoken about openly…the quality of the information online is very poor and often comes across as just someone’s personal opinion*”

### Level of knowledge

**Fig. 2:**
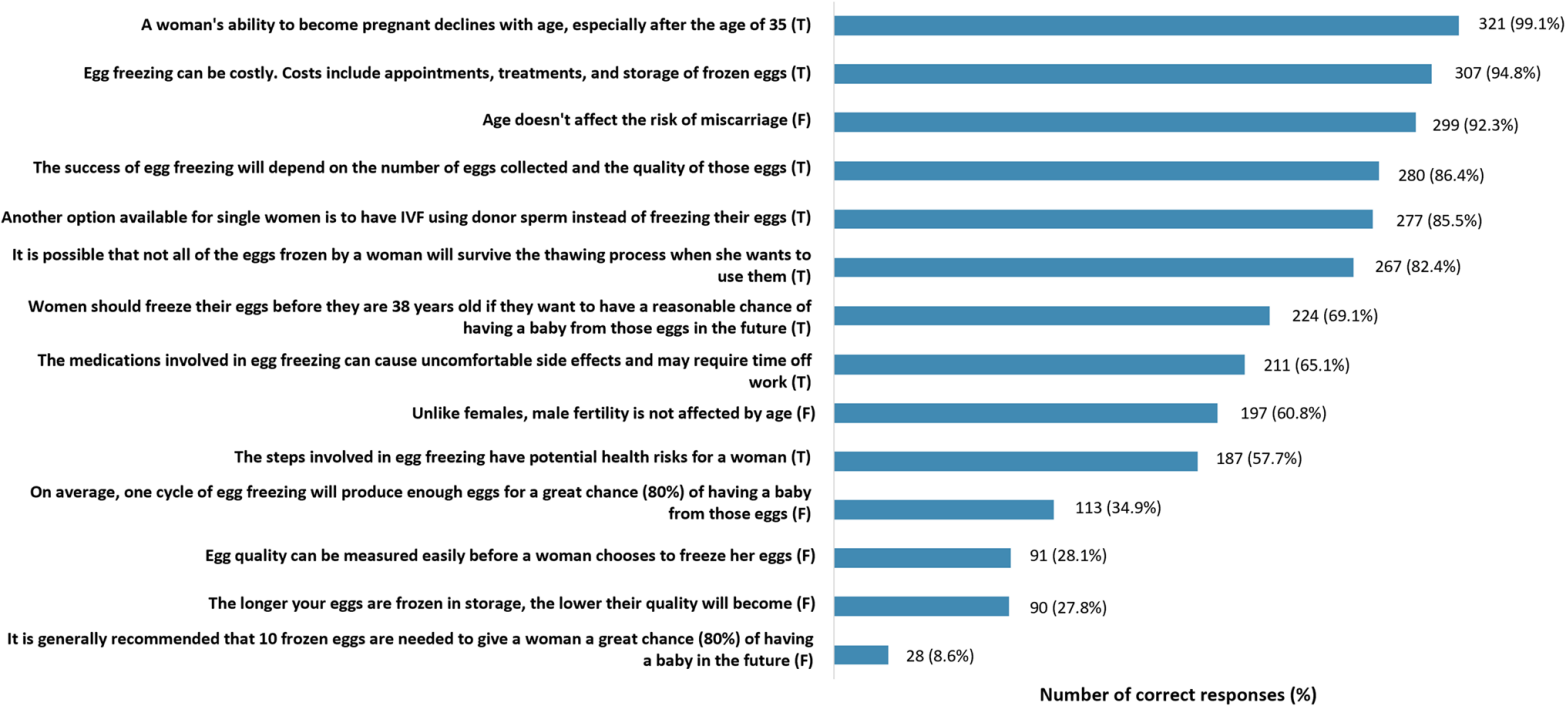
Number (%) of correct responses for knowledge questions amongst survey participants. Questions were reordered by the number of correct responses. Results are based on the number of non-missing responses for each knowledge question (*n*=324). (T) = True, (F) = False

Mean knowledge score was 8.9/14 (SD: 2.3) (Table 3), indicating a moderate understanding of POC and age-related infertility. Five of the 14 knowledge questions were answered with incorrect or unsure responses by >40% of participants. These questions related to POC procedure associated health-risks, success rates, limitations to assessing oocyte quality before collection, and whether oocyte quality reduces with time in storage (Fig. 2).

### Decisional conflict

For participants who had considered POC (*n*=249), mean DCS score was 57.1/100 (SD: 27.2), and most (78%) had high decisional conflict (score >37.5) (Table 5).

**Table 5:** Decisional conflict and time to decision amongst those who had considered planned oocyte cryopreservation

	Number (%)
Decisional Conflict Scale	
Total score (out of 100), mean (SD) (*n*=235)	57.1 (27.2)
Categories (*n*=235)	
Low decisional conflict (score <25)	31 (13.2%)
Moderate decisional conflict (score 25-37.5)	21 (8.9%)
High decisional conflict (score >37.5)	183 (77.9%)
Sub-scale scores (out of 100), median (IQR)	
Uncertainty (*n*=237)	100.0 (50.0-100.0)
Uninformed (*n*=238)	66.7 (33.3-100.0)
Unclear about personal values (*n*=236)	50.0 (25.0-100.0)
Unsupported in decision making (*n*=238)	33.3 (16.7-66.7)
Time to Decision	
Months spent considering POC, median (IQR) (*n*=210)	12.0 (6.0-24.0)
Months spent considering POC by undecided participants, median (IQR) (*n*=168)	12.0 (6.0-24.0)
Months spent considering POC by decided participants, median (IQR) (*n*=42)	24.0 (12.0-36.0)

Data are presented as *n* (%) unless otherwise stated. POC = Planned Oocyte Cryopreservation, DCS = Decisional Conflict Scale, SD = Standard deviation, IQR = 25th to 75th percentile

Participants included in the multivariable linear regression model had similar characteristics to the entire cohort that had considered POC (Supplementary Table 1). Factors omitted from the model were: language spoken at home (*p*=0.33); education level (*p*=0.80); medical or health-related education (*p*=0.41), and; number of existing children (*p*=0.35). Participants who had searched for POC information had a lower mean DCS score than those who had not (-19.3 [95% CI]: (-26.3, -12.2), *p*<0.001), however, this variable was also not included in the model due to high collinearity with consulting an IVF specialist. In the multivariable linear regression model, mean DCS score was lower for participants who had consulted an IVF specialist vs had not (-17.5, 95% CI [-28.0, -7.1], *p*=0.001), and with every 1-point increase in knowledge score (-2.4, 95% CI [-3.9, -0.8], *p*=0.003). Participants who decided, either to freeze oocytes (-33.9, 95% CI [-49.1, -18.8], *p*<0.001) or not to freeze oocytes (-10.3, 95% CI [-20.4, -0.2], *p*=0.045), had lower mean DCS scores than those who were undecided. When comparing POC uptake, mean DCS score was lower for participants who chose to freeze their oocytes vs those who decided against it (-23.6, 95% CI [-40.2, -7.0], *p*=0.005) (Table 6).

**Table 6:** Linear regression analysis of decisional conflict scale scores amongst those who had considered planned oocyte cryopreservation

		Univariable Analysis^‡^		Multivariable Analysis^§^	
Characteristic	*N*, Mean DCS (SD)	Estimate (95% CI) Univariable	*P* value	Estimate (95% CI) Multivariable	*P* value
Age*					
≤ 25 years	61, 58.0 (24.4)	Reference		Reference	
26 to ≤ 30 years	52, 61.2 (25.7)	3.2 (-6.8, 13.2)	0.53	8.9 (0.1, 17.6)	0.047
31 to ≤ 35 years	45, 59.8 (25.2)	1.7 (-8.7, 12.2)	0.74	7.2 (-2.0, 16.4)	0.13
36 to ≤ 40 years	31, 46.5 (35.2)	-11.6 (-23.3, 0.1)	0.05	5.4 (-5.7, 16.6)	0.34
> 40 years	8, 36.9 (24.0)	-21.2 (-41.1, -1.2)	0.038	-8.7 (-26.6, 9.2)	0.34
*Relationship status*					
Single	99, 57.8 (27.2)	Reference		Reference	
In a committed relationship and living together, engaged, married or de facto	80, 57.2 (27.8)	-0.6 (-8.5, 7.3)	0.89	0.4 (-7.0, 7.8)	0.91
In a committed relationship but not living together	38, 53.7 (25.0)	-4.1 (-14.2, 5.9)	0.42	-4.3 (-14.2, 5.5)	0.38
In a relationship but not committed	11, 73.6 (20.1)	15.8 (-0.9, 32.6)	0.06	10.9 (-5.9, 27.8)	0.20
Separated/divorced	6, 28.3 (26.4)	-29.5 (-51.6, -7.3)	0.009	-12.3 (-45.9, 21.3)	0.47
*Consulted an IVF specialist*					
No	190, 62.9 (23.5)	Reference		Reference	
Yes	40, 30.6 (28.5)	-32.3 (-40.7, -23.9)	<0.001	-17.5 (-28.0, -7.1)	0.001
*Decision outcome/uptake of POC †*					
Undecided	185, 62.7 (23.2)	Reference		Reference	
Decided to freeze	15, 13.7 (23.9)	-49.0 (-61.8, -36.3)	<0.001	-33.9 (-49.1, -18.8)	<0.001
Decided not to freeze	35, 46.0 (28.8)	-16.7 (-25.5, -7.9)	<0.001	-10.3 (-20.4, -0.2)	0.045
*Knowledge score*					
1-point increase	235, 57.1 (27.2)	-3.4 (-4.9, -1.9)	<0.001	-2.4 (-3.9, -0.8)	0.003

DCS = Decisional Conflict Scale, SD = Standard Deviation, CI = Confidence Interval, IVF = In-Vitro Fertilisation, POC = Planned Oocyte Cryopreservation

^*^Age was categorised into five-year groupings due to nonlinearity with DCS

^†^Mean DCS score for decided participants (i.e. those who decided to freeze or not to freeze their oocytes) is 36.3 (*n*=50, SD: 31.0). The adjusted estimate and 95% CI for decided participants vs undecided participants is -18.4 (-27.5, -9.3), *p*<0.001. The adjusted estimate and 95% CI for participants who decided to freeze their oocytes vs participants who decided not to freeze oocytes is -23.6 (-40.2, -7.0), *p*=0.005

^‡^Covariates presented are those with *p*<0.20 in the univariable linear regression model

^§^Covariates with *p*<0.05 in the multivariable model are: consulted an IVF specialist (*p*=0.001), decision outcome/uptake of POC (*p*<0.001), and knowledge score (*p*=0.003). Sample used in the multivariable linear regression model (*n*=191) were participants who had considered POC and provided data for all included variables

### Time to decision

Median time to decision for decided participants (*n*=53, 16%) was 24-months (IQR: 12-36, Table 5). Of the participants who had considered POC, 53 (25%) spent ≤6 months; 55 (26%) spent 7-12 months, 60 (29%) spent 13-24 months, 36 (17%) spent 25-60 months, and 6 (3%) spent >60 months contemplating its use. In an ordered logistic regression model, a 5% relative increase in the odds of experiencing prolonged indecision was associated with every 5-point lower DCS score (odds ratio: 1.05, 95% CI [1.01, 1.10], *p*=0.02). This suggests that participants with lower DCS scores were more likely to have considered POC for longer than participants with higher DCS scores.

## Discussion

This novel study aimed to identify the information and decision support needs of reproductive aged women who were interested in receiving POC information. Overall, participants had gaps in their understanding of POC highlighting a need to improve awareness. The majority had considered POC but only about half had searched for information about it. For these participants, the predominant information source was fertility clinic websites. Few sought clinical advice. Many of the women who had considered POC had high decisional conflict, indicating a need for decision support. Those who reached a POC decision generally spent around two-years deciding. There was also strong support for providing women POC information by age 30 years. Preferred information providers were healthcare professionals or online resources.

Similar to previous reports of POC users, most participants were single, childless, university educated, and working in professional occupations [11, 22, 45, 46]. Unexpectedly, half had medical or other health-related training. Comparable data from similar studies are limited as this type of education is often not reported. One study of 150 POC users from the US and Israel showed 16% had a medical degree [47], however our cohorts are not directly comparable. It is possible that women working in these professions have a greater interest in POC as the challenges of career progression, particularly in the medical field, can often influence the timing of parenthood [48–50], and hence consideration of use. Whilst career advancement is not considered a strong motivator for POC use [11], these women may still consider the option even if it does not translate to uptake.

Time to decision was typically around two-years for those who had decided about POC. This is consistent with data showing that most childless reproductive aged women remained undecided about POC after a two-year period [30]. The average age at POC in published data to date is mid-late 30s [1, 2, 11, 15]. A two-year contemplation period for women in their mid-30s or above is likely to reduce the benefit of POC and ultimately live birth outcomes [1]. Previous authors suggested that women become more engaged in POC decisions as they approach the end of their reproductive years [30]. However, most of our participants believed that women should be informed about the option by age 30 years suggesting a desire to engage in earlier decision-making. Some women regret not freezing their oocytes earlier [27, 51]. Therefore, informing women about POC at a younger age may support better pregnancy planning, and potentially timelier uptake. Nonetheless, barriers to access POC, such as costs and storage time limits [10, 11, 52], may still prevent women from using the technology at more ideal age (e.g. in their 20s or early 30s) [1, 2]. In addition, earlier consideration of POC may encourage more women to freeze oocytes which ultimately are never used [17]. Thus, it may also expose more women to unnecessary health risks, costs, and future decisions about the disposition of their oocytes. Women should be provided with comprehensive information at the time of considering POC to facilitate informed choice. This includes information about frozen oocyte usage rates and the disposition of unused oocytes.

Most participants believed healthcare professionals should provide POC information, demonstrating a need for clinical, evidence-based advice. Fertility specialists were the preferred source. However, if women want to engage in early education about POC, primary care physicians (e.g. general practitioners) may be best placed to initially discuss the option. Research into primary care physicians providing POC information is limited. However, these clinicians may find it challenging to discuss the topic and fertility more broadly, due to limited knowledge, appointment time constraints, and concerns about causing distress and appearing paternalistic or presumptuous towards their patients [36, 53, 54]. A small Australian study (*n*=72) reported that most primary care physicians wanted more information and training about POC, indicating a willingness to assist patients with these decisions [36]. This type of educational support could be provided by professional societies [36]. Although some patients may find it inappropriate for primary care physicians to provide unprompted fertility-related information [54], many others approve of them raising the topic [55]. To navigate these conversations, primary care physicians could ask their patients if they are interested in discussing their reproductive intentions. Suitable times for these conversations may be during general or reproductive health appointments [11, 56]. Also, despite most of our participants believing POC information should be provided by primary care physicians, few had consulted them consistent with previous data [56].

Over half our participants had searched for POC information mainly from fertility clinic websites. In addition, online information delivery was rated as the most useful method to support POC decisions. Together, this highlights the value of providing POC information online. Web-based materials are private, highly accessible, and do not require attending clinic appointments which usually incur costs and have restricted hours of access.

Also, our data suggests that some women make their decision (particularly against POC) without obtaining clinical advice. This is different to other health-related treatment decisions where clinicians are involved in the decision from the outset [57]. Specialist counselling is necessary for those who wish to proceed with POC or want personalised information to consider [58, 59]. But, for a subset of women who ultimately decide against POC, general information alone may be sufficient to inform their decision. In addition to receiving POC information from primary care physicians, an online resource providing evidence-based, unbiased information about POC and its alternatives, may support decision-making including whether to consult an IVF specialist.

Most participants who considered using POC had high decisional conflict, demonstrating the difficulty of this decision. Higher DCS scores are associated with poorer knowledge of options, greater decision regret, and decision delay [39, 60]. In our study, consulting an IVF specialist about POC was highly associated with lower decisional conflict. However, given many women have high decisional conflict even after receiving specialist counselling [26], there appears to be a need for additional support to supplement clinical advice. A Decision Aid for POC may reduce decisional conflict as demonstrated in other health areas [61]. These tools are used to support shared decision-making [62, 63] and are recommended by the European Society of Human Reproduction and Embryology for fertility preservation decisions [52]. There are Decision Aids for other elective procedures [61] including the use of fertility preservation for medical reasons [64, 65]. The International Patient Decision Aid Standards Collaboration [66] provide a framework which can be implemented to develop a Decision Aid for POC. Finally, we found no association between high decisional conflict and advanced age (>37 years) as demonstrated in prior research [26]. This may reflect population differences between the two studies.

Participants who decided against POC had higher decisional conflict than those who decided to freeze their oocytes. This may reflect a difference in the information resources used by the two groups. For instance, most women who chose to freeze their oocytes received specialist counselling, whilst only a few who decided against POC sought similar advice, and consequently may be less informed. It is possible that barriers to access POC, such as affordability [10, 11], could mean that some women decide POC is unfeasible early in the consideration process without conducting full investigations into the option.

Overall, participants were moderately informed about POC and age-related infertility. Low knowledge of these concepts was reported in the general population using a similar knowledge scale [14]. The difference in comprehension likely reflects our cohort’s interest in the topic and prior research performed by some participants. Main knowledge gaps related to POC procedure associated health risks, success rates, limitations to assessing oocyte quality, and whether time in storage reduced oocyte quality. In addition, we observed an association between higher knowledge score and lower decisional conflict. Together, this suggests that addressing these gaps in knowledge, through specialist counselling or other educational initiatives, may reduce POC decisional conflict. The provision of accurate, transparent, and accessible information about POC success rates is particularly important as IVF success rates are often overestimated [67–69]. It is possible that some women may choose to defer their decision about POC because they misjudge its potential benefit at an older age, which could create unrealistic expectations about the technology when they return to their decision in the future.

This is the first study to our knowledge measuring decisional conflict and time to decision in a broad, community-based group of women who have considered using POC. It addresses a gap in evidence about the information and decision support needs of those who want to receive POC information. The generalisability of these findings may be limited, although the extent of these limitations is difficult to determine as data from a directly comparable group are not available. It is possible that women were more likely to participate in this study if they were actively considering POC or searching for information. Participants may also reflect women with higher decisional conflict than in the population. However, given the only other study reporting on POC decisional conflict also found that many women who received specialist counselling had high DCS scores [26], this may not be the case. Whilst we aimed to recruit women from the community who were interested in receiving POC information, we observed an unexpectedly high proportion of participants with medical or health-related education. It is possible these women are more likely to consider POC, but it may also reflect the recruitment methods used. In addition, we observed participant attrition (Fig. 1). Participants with a greater interest in POC may have been more likely to complete the survey. We were ethically obliged to use data from incomplete surveys as participants had spent time providing us their information and did not withdraw from the study. This has contributed to some missing data observed in our analysis, however, question completion rates were high (>82%) (Supplementary Table 2). Other limitations inherent to any cross-sectional survey are the use of self-reported data, and the relevancy of results over time particularly in the rapidly evolving field of POC. Our knowledge scale was designed in the absence of a relevant validated scale; hence it may not measure actual knowledge as intended. The scale aims to assess understanding of general concepts and does not consider nuances that may arise with individuals or changes to success rates over time.

## Conclusion

Women interested in receiving POC information had gaps in their knowledge of the topic including its potential health risks and success rates. They also believed women should receive POC information by age 30 years, preferably by healthcare professionals or online resources. Most who had considered using POC had high decisional conflict demonstrating a need for decision support. Time to decision was typically around two-years which may impact the chances of a successful pregnancy from frozen oocytes in the future. There is a need for comprehensive and transparent information, and decision support to help women make informed and timely POC decisions. Primary care physicians and online resources may help to address women’s gaps in knowledge, reduce their decisional conflict, and encourage earlier consideration of POC.

## Supplementary information

ESM 1 (DOCX 124 kb)

## Data Availability

The datasets and materials used in this study may be available from the corresponding author upon reasonable request.
