# Supplementary material for: Information and decision support needs: A survey of women interested in receiving planned oocyte cryopreservation information
Source: J Assist Reprod Genet. 2023 Apr 14;40(6):1265–80. doi: 10.1007/s10815-023-02796-x (PMC10101825; doi:10.1007/s10815-023-02796-x)
Supplement: Supplementary file 1 — (DOCX 124 kb) [file 10815_2023_2796_MOESM1_ESM.docx]

**Supplementary Table 1: Characteristics of all participants who considered Planned Oocyte Cryopreservation vs those included in the multivariable linear regression model**

|  | **Considered POC (n=249)** | **Considered POC and included in the linear regression multivariable analysis model (n=191)*** |
| --- | --- | --- |
| **Age** |  |  |
| ≤ 25 years | 66/210 (31.4%) | 59/191 (30.9%) |
| 26 to ≤ 30 years | 56/210 (26.7%) | 50/191 (26.2%) |
| 31 to ≤ 35 years | 46/210 (21.9%) | 44/191 (23.0%) |
| 36 to ≤ 40 years | 33/210 (15.7%) | 30/191 (15.7%) |
| > 40 years | 9/210 (4.3%) | 8/191 (4.2%) |
| **Relationship Status** |  |  |
| Single | 106/248 (42.7%) | 85/191 (44.5%) |
| In a committed relationship and living together, engaged, married or de facto | 83/248 (33.5%) | 66/191 (34.6%) |
| In a committed relationship but not living together | 40/248 (16.1%) | 30/191 (15.7%) |
| In a relationship but not committed | 13/248 (5.2%) | 8/191 (4.2%) |
| Separated/divorced | 6/248 (2.4%) | 2/191 (1.0%) |
| **Relationship length for partnered participants** |  |  |
| <1 year | 22/133 (16.5%) | 18/101 (17.8%) |
| 1 year to ≤ 5 years | 69/133 (51.9%) | 54/101 (53.5%) |
| >5 years | 42/133 (31.6%) | 29/101 (28.7%) |
| **Location: Australian state or territory** |  |  |
| New South Wales | 53/245 (21.6%) | 40/188 (21.3%) |
| Victoria | 127/245 (51.8%) | 98/188 (52.1%) |
| Other states/territories | 65/245 (26.5%) | 50/188 (26.6%) |
| **Location: Rural, remote or metropolitan area** |  |  |
| Metropolitan | 190/245 (77.6%) | 149/188 (79.3%) |
| Metropolitan/rural border | 10/245 (4.1%) | 7/188 (3.7%) |
| Rural | 45/245 (18.4%) | 32/188 (17.0%) |
| **Years living in Australia** |  |  |
| < 10 years | 17/216 (7.9%) | 14/173 (8.1%) |
| ≥ 10 years | 199/216 (92.1%) | 159/173 (91.9%) |
| **Aboriginal or Torres Strait Islander descent** |  |  |
| No | 241/248 (97.2%) | 183/190 (96.3%) |
| Yes | 7/248 (2.8%) | 7/190 (3.7%) |
| **Language spoken at home** |  |  |
| English | 239/249 (96.0%) | 184/191 (96.3%) |
| Other | 10/249 (4.0%) | 7/191 (3.7%) |
| **Highest level of education completed** |  |  |
| High school | 21/249 (8.4%) | 20/191 (10.5%) |
| Trade (TAFE) certificate/diploma | 26/249 (10.4%) | 16/191 (8.4%) |
| Bachelor degree | 98/249 (39.4%) | 76/191 (39.8%) |
| Postgraduate diploma/degree | 104/249 (41.8%) | 79/191 (41.4%) |
| **Studied in a medical or other health-related field** |  |  |
| No | 130/249 (52.2%) | 100/191 (52.4%) |
| Yes | 119/249 (47.8%) | 91/191 (47.6%) |
| **Employment status** |  |  |
| Full-time employed | 155/249 (62.2%) | 116/191 (60.7%) |
| Part-time employed | 52/249 (20.9%) | 40/191 (20.9%) |
| Full-time student | 30/249 (12.0%) | 25/191 (13.1%) |
| Unemployed | 7/249 (2.8%) | 5/191 (2.6%) |
| Other | 5/249 (2.0%) | 5/191 (2.6%) |
| **Occupation** |  |  |
| Professional | 194/248 (78.2%) | 147/190 (77.4%) |
| Full time student | 30/248 (12.1%) | 25/190 (13.2%) |
| Other | 24/248 (9.6%) | 18/190 (9.5%) |

Data are presented as n/total (%). *Participants were included in the multivariable regression analysis if data were available for all dependent and independent variables. POC = ‘Planned Oocyte Cryopreservation’.

**Supplementary Table 2: Survey question completion rate**

| **Question** | **Number of responses (%)** |
| --- | --- |
| **Participant Characteristics** |  |
| Age | 274 (82.5%) |
| Relationship status | 331 (99.7%) |
| Relationship length | 193 (95.5%)^*^ |
| Location | 327 (98.5%) |
| Years living in Australia | 287 (86.4%) |
| Aboriginal or Torres Strait Islander descent | 331 (99.7%) |
| Language spoken at home | 332 (100%) |
| Highest level of education completed | 332 (100%) |
| Studied in a medical or other health-related field | 332 (100%) |
| Employment status | 332 (100%) |
| Occupation | 331 (99.7%) |
| Number of existing children | 331 (99.7%) |
| Stage of considering POC | 332 (100%) |
| Desire to have (more) children in the future | 331 (99.7%) |
| Timing to have (more) children in the future | 224 (100%)^†^ |
| Reason for interest in POC | 332 (100%) |
| **Knowledge Scale** |  |
| A woman's ability to become pregnant declines with age, especially after the age of 35. | 324 (97.6%) |
| Age doesn't affect the risk of miscarriage. | 324 (97.6%) |
| Women should freeze their eggs before they are 38 years old if they want to have a reasonable chance of having a baby from those eggs in the future. | 324 (97.6%) |
| It is generally recommended that 10 frozen eggs are needed to give a woman a great chance (80%) of having a baby in the future. | 324 (97.6%) |
| On average, one cycle of egg-freezing will produce enough eggs for a great chance (80%) of having a baby from those eggs. | 324 (97.6%) |
| The medications involved in egg-freezing can cause uncomfortable side effects, and may require time off work. | 324 (97.6%) |
| Another option available for single women is to have IVF using donor sperm instead of freezing their eggs. | 324 (97.6%) |
| The steps involved in egg freezing have potential health risks for a woman. | 324 (97.6%) |
| The success of egg-freezing will depend on the number of eggs collected and the quality of those eggs. | 324 (97.6%) |
| The longer your eggs are frozen in storage, the lower their quality will become. | 324 (97.6%) |
| Egg quality can be measured easily before a woman chooses to freeze her eggs. | 324 (97.6%) |
| Egg freezing can be costly. Costs include appointments, treatments, and storage of frozen eggs. | 324 (97.6%) |
| It is possible that not all of the eggs frozen by a woman will survive the thawing process when she wants to use them. | 324 (97.6%) |
| Unlike females, male fertility is not affected by age. | 324 (97.6%) |
| **Decisional Conflict Scale** |  |
| Do you know which options are available to you? | 239 (96.0%)^‡^ |
| Do you know the benefits of each option? | 238 (95.6%)^‡^ |
| Do you know the risks and side effects of each option? | 238 (95.6%)^‡^ |
| Are you clear about which benefits matter to you the most? | 238 (95.6%)^‡^ |
| Are you clear about which risks and side effects matter to you the most? | 236 (94.8%)^‡^ |
| Do you have enough support from others to make a decision? | 238 (95.6%)^‡^ |
| Are you choosing without pressure from others? | 238 (95.6%)^‡^ |
| Do you have enough advice to make a choice? | 238 (95.6%)^‡^ |
| Are you clear about the best choice for you? | 237 (95.2%)^‡^ |
| Do you feel sure about what to choose? | 238 (95.6%)^‡^ |
| **Information Resources** |  |
| Methods used to research POC | 301 (90.7%) |
| Consulted an IVF specialist | 302 (91.0%) |
| Spoke to friends about POC | 299 (90.1%) |
| Time spent searching for POC information | 301 (90.7%) |
| **Preferences for Information Delivery** |  |
| What age women should be informed about POC | 292 (88.0%) |
| Preferred providers of POC information | 300 (90.4%) |
| Usefulness of different information delivery methods | 290 (87.3%) |
| **Time to Decision** |  |
| Length of time spent considering POC | 210 (84.3%)^‡^ |

POC = ‘Planned Oocyte Cryopreservation’. IVF = ‘In-Vitro Fertilisation’. Denominator is 332 unless otherwise stated. *Conditional question based on relationship status. Denominator (n=202) excludes single participants. ^†^Conditional question based on desire for (more) children. Denominator (n=224) excludes participants who reported not wanting to have (more) children or were unsure. ^‡^ Denominator (n=249) excludes participants who had not considered POC before.
